# The effects of prophylactic cranial irradiation versus control on survival of patients with extensive-stage small-cell lung cancer: a meta-analysis of 14 trials

**DOI:** 10.1186/s13014-018-1101-3

**Published:** 2018-08-23

**Authors:** Wei Ge, Huilin Xu, Yafei Yan, Dedong Cao

**Affiliations:** 1 Department of Oncology, RenMin Hospital of WuHan University, WuHan, 430000 Hubei China; 2 Department of Oncology, The Fifth Hospital of WuHan, WuHan, 430000 Hubei China

**Keywords:** Small-cell lung cancer, Prophylactic cranial irradiation, Survival, Meta-analysis, Brain metastasis, Evidence-based medicine

## Abstract

**Background:**

A recent Japanese study suggested prophylactic cranial irradiation (PCI) failed to improve survival of extensive-stage small-cell lung cancer (SCLC). However, previous studies showed that PCI was beneficial in reducing the rate of mortality for extensive-stage SCLC. In this study, we aimed to evaluate the impact of PCI on the survival of patients diagnosed with extensive-stage SCLC by meta-analysis.

**Methods:**

PubMed, Embase, the Cochrane library and Chinese Biomedical Literature database (CBM) were systematically searched to identify eligible clinical studies assessing the efficacy of PCI in extensive-stage SCLC patients. After extracting survival data, brain metastasis, and response rates, the pooled estimates were calculated.

**Results:**

A total of 14 clinical studies were included, involving 1221 cases in the PCI group and 5074 in the control group. The results showed that PCI significantly improved overall survival (Hazard ratio (HR) = 0.57; 95% confidence interval (CI): 0.47, 0.69; *p* < 0.001) and brain metastasis (risk ratio (RR) =0.47, 95%CI: 0.33, 0.69; *p* < 0.01). Subgroup analysis along with sensitivity analysis suggested that PCI effects on overall survival were independent of region, pre-PCI brain metastasis status and PCI administration timing.

**Conclusion:**

PCI improves overall survival in extensive-stage SCLC. More randomized controlled trials are needed to verify our findings.

**Electronic supplementary material:**

The online version of this article (10.1186/s13014-018-1101-3) contains supplementary material, which is available to authorized users.

## Background

Small cell lung cancer (SCLC) accounts for 13% to 20% of lung cancer, and 60% to 70% of SCLC patients have extensive disease at the time of initial diagnosis [1, 2]. Platinum-based chemotherapy is one of the main treatments for extensive-stage SCLC [2]. The prognosis is poor, with a median survival of less than 1 year [3]. The propensity of brain metastasis (BM) is one major difference between SCLC and other lung cancers. About 10% of SCLC patients experience BM at diagnosis and it rises to 50% or even higher after 2-year survival [4]. It is demonstrated that there is a positive role of prophylactic cranial irradiation (PCI) on survival in resected [5] and limited-stage SCLC patients [6]. Whether PCI should be used for extensive-stage SCLC is still in debate.

In 2007, a randomized trial reported the role of PCI in extensive-stage SCLC patients who responded to chemotherapy [7]. The primary endpoint was symptomatic brain metastases time. The results showed that the risk of symptomatic brain metastases in PCI group was as low as 0.27 times compared to that of the control group, along with a prolonged overall survival (OS) from 5.4 months to 6.7 months [7]. These findings suggested PCI was useful in reducing BM risk and prolonging survival [7]. Since then, several retrospective studies [8, 9] also evaluated the effects of PCI on survival and incidence of BM and showed similar results. However, Toshiaki et al. [10] showed that PCI did not significantly improve OS in extensive-stage SCLC. They reported that the median OS of PCI versus control was 11.6 months versus 13.7 months (HR = 1.27, *p* = 0.094). Therefore, they concluded that PCI was not essential for extensive-stage SCLC, regardless of prior response to chemotherapy [10]. These conflicting results contradict previous findings that highlighted a positive effect of PCI on extensive-stage SCLC.

To answer whether PCI could have a beneficial effect on OS in extensive-stage SCLC, we collected clinical studies assessing the role of PCI versus control in patients with extensive-stage SCLC. Survival data were combined to determine the impact of PCI versus control on the survival of extensive-stage SCLC. The pooled results suggest that PCI could benefit these patients by prolonging survival.

## Methods

This study was performed based on the PRISMA and the Cochrane handbook guidelines [11].

### Systematic search

Electronic databases including PubMed, Embase, the Cochrane library, and the Chinese Biomedical Literature Database were systematically searched with deadline on March 2018. Search terms were “prophylactic cranial irradiation or PCI”, “lung carcinoma or lung cancer or lung tumor or lung neoplasm”, “extensive or advanced”. These terms were used in different combinations without language restrictions.

### Inclusion and exclusion criteria

Inclusion criteria: (1) Patients were diagnosed by pathology or cytology to confirm SCLC, and had imaging exams to determine extensive-stage disease; (2) OS data of extensive-stage SCLC were reported; (3) If results from the same patient population were published in different journals, the most complete or up-to-date study was selected; (4) Eligible studies were not only published full-text but also abstract, conference meeting presentation and unpublished literature.

Exclusion criteria: Studies were excluded if they met the following (1) Insufficient data on survival; (2) Limited-stage SCLC, non-small cell lung cancer, or other metastatic tumor; (3) Review, animal experiments, comments, survey, and guidelines.

### Data extraction

Two researchers conducted the screening of eligible studies, independently. When there was an inconsistency, it was solved through discussion. The following information was extracted: first author, time of publication, region, participant number, sex, age, received chemotherapy or not before PCI, PCI administration timing, thoracic radiotherapy, confirmed absence of brain metastases before PCI (pre-PCI BM status), and survival and efficacy data.

### Quality assessment

The Newcastle-Ottawa scale (NOS) method [12] was used to assess the quality of the included retrospective studies. Quality evaluation of randomized studies was performed using the literature quality assessment method provided by the Cochrane Handbook 5.1. [11] The NOS focuses on three aspects during evaluation: the selection of the cohort, the comparability of the cohort, and the evaluation of the results. A maximum of 4 stars can be obtained in the selection, and the comparability can be obtained by up to 2 stars. The evaluation of the results can be up to 3 stars. The highest quality is assigned the highest number of stars. In the Cochrane Handbook approach, each randomized study is evaluated in five major aspects: Random, Allocation, Blinding, Selective reporting, and Other bias.

### Statistical method

The meta-analysis was used to combine the individual HRs and its related 95% CIs to draw a forest plot. Q test and I^2^ statistic were used to test the heterogeneity across the included studies. When *p* < 0.1 or I^2^ > 50%, it suggested a significant heterogeneity between the included trials. Then the random effects model was used; when *p* > 0.1 or I^2^ < 50%, it indicated no significant heterogeneity and the results were combined using a fixed effect model [11].

The HRs of PCI versus control on OS were extracted. If there was only a survival curve comparing PCI versus control without HRs values, the reported methods [13, 14] were used to calculate the HRs. Combined HR < 1 indicated that PCI was a favorable factor for extensive-stage SCLC, whereas HR > 1 indicated the opposite. BM incidences after PCI versus control were also extracted to determine the influence of PCI on the risk of BM. Subgroup analyses of OS were performed in terms of study type, region, and timing of PCI administration. The funnel plot, Begg’s method, and Egger’s method were used to detect the publication bias, and *P* < 0.05 suggested there was a significant publication bias [15, 16]. Sensitivity analysis was performed to confirm the stability of the results if necessary. Meta-analysis was performed using RevMan 5.3 and Stata 11 software. For a combined estimate, a *P* < 0.05 was considered as there was a statistical significance.

## Results

### Search results

**Fig. 1:**
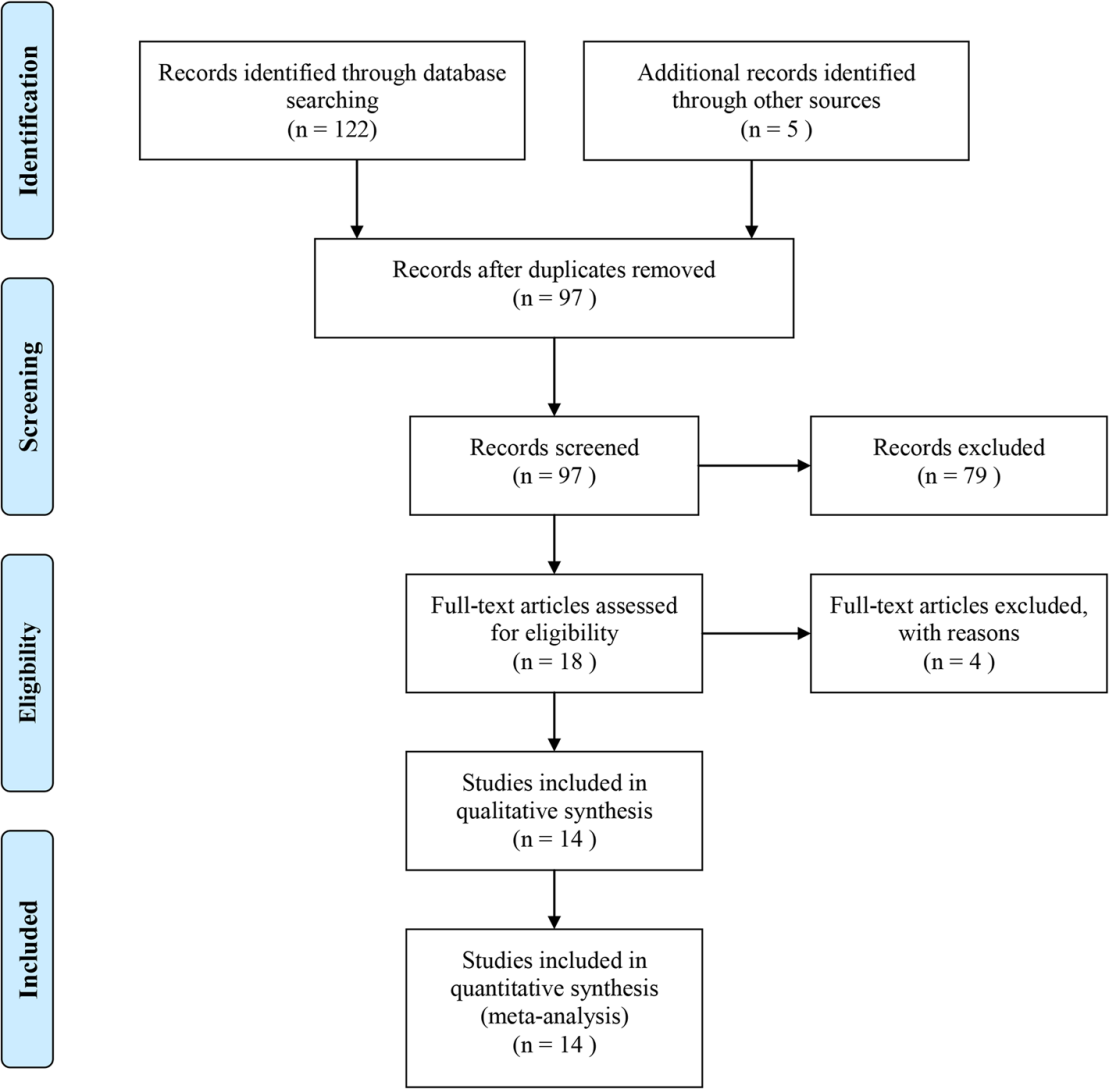
Flow-chart of identifying eligible studies

A total of 127 studies were retrieved through the comprehensive search,. After reading the title and abstract, 109 of them were excluded due to duplication, review, animal study and other reasons, and the remaining 18 articles were further reviewed. After reading the full-text, 14 articles met the inclusion criteria and were considered as eligible studies [7–10, 17–26]. The details of the literature search process are shown in Fig. 1.

### Baseline characteristics of included studies

A total of 6295 patients with extensive-stage SCLC were included, involving 1221 patients in the PCI group and 5074 patients in the control group. These studies reported the impact of PCI on overall survival. If the literature provided the univariate and multivariate analysis results of overall survival, the multivariate analysis results were used because they minimized the influence of confounding factors. Eight [7–10, 18, 20, 24, 25] of included studies provided HRs and related 95% CI. HRs were also obtained based on survival curves from five retrospective trials [17, 21–23, 26]. The basic characteristics of each study are shown in Table 1.

**Table 1:** Baseline characteristics of included studies

Authors	Year	N	Age		Sex(M/F)	Chemotherapy		PCI		Thoracic radiotherapy		Brain metastasis		Outcomes
			PCI	Control		regimen	Y/N	Y	N	Y	N	Y	N	
Shu	2016	46	60.4		35/11	EP/IP	46/0	6	40	20	26	11	35	SR, OS, ORR
Huang	2015	73	NR		60/13	NR	70/3	20	53	30	43	24	49	SR, OS
Bai	2015	188	59 (22–81)		115/73	EP	NR	110	78	107	81	NR	NR	SR, OS
Wu	2015	302	61 (34–81)		212/43	EP/EC	219/56	34	241	109	166	60	215	SR, OS, ORR
Salama	2016	85	60 (39–77)		38/47	Sunitinib	44/41	41	44	NR	NR	NR	NR	SR, OS, ORR
Nicholls	2016	129	65.4		NA	EC	101/28	43	86	63	66	39	90	OS, DFS
Rule	2015	71	73 (70–80)		NA	EP	71/0	27	44	NA	NA	NR	NR	OS, ORR
Schild	2012	318	62		NA	EP	318/0	124	194	NA	NA	NR	NR	OS, ORR
Sharma	2017	4257	NR		2065/2192	standard chemotherapy	4257/0	473	3784	NR	NR	0	4257	OS, ORR
Slotman	2007	286	62 (37–75)	63 (39–75)	179/107	standard chemotherapy	286/0	143	143	NR	NR	0	286	SR, OS, DFS
Toshiaki	2017	224	69 (43–83)	69 (37–86)	193/31	platinum-based chemotherapy	224/0	113	111	NR	NR	0	224	OS, ORR
Chen	2016	204	57 (50–62)	59 (53–64)	171/33	platinum-based chemotherapy	204/0	45	159	NR	NR	17	187	OS, ORR
Wang	2014	93	61 (33–82)		74/19	EP	90/3	26	67	56	37	50	43	SR, OS, ORR
Adriana	2018	46	65 (42–81)		27/19	platinum-based chemotherapy	46/0	16	30	2	44	0	46	OS, PFS

*Abbreviation*: *N* number, *PCI* Prophylactic cranial irradiation, *M* male, *F* female, *Y* yes, *N* no, *EP* etoposide and cisplatin, *IP* Irinotecan and cisplatin, *EC* etoposide and carboplatin, *SR* survival rate, *OS* overall survival, *ORR* overall response rate, *NR* not reported, *NA* not available, *DFS* disease free survival, *PFS* progression free survival

### Quality assessment

Two randomized trials [7, 10] were included, so the quality assessment method of the Cochrane Handbook 5.1 was used. As shown in Table 2, these two studies had a low risk of bias and were considered as high-quality studies. As most of the included trials were retrospective studies, the NOS method [12] was applied. All included studies were considered as moderate or high quality (Table 2). The main reasons for lowering the overall quality were selection and outcome bias.

**Table 2:** Assessment of quality among included studies

Studies	Year	Random	Allocation	Blinding	Selective reporting	Other bias
Slotman	2007	Y	N	N	N	NR
Toshiaki	2017	Y	N	N	N	NR
		Selection		Comparability	Outcome	
Shu	2016	★★		★	★★	
Huang	2015	★★		★★	★★	
Bai	2015	★★★		★	★★	
Wu	2015	★★★		★★	★★★	
Salama	2016	★★★		★★	★★★	
Nicholls	2016	★★		★★	★★	
Rule	2015	★★		★★	★★	
Schild	2012	★★★		★★	★★★	
Sharma	2017	★★★		★★	★★★	
Chen	2016	★★★		★★	★★★	
Wang	2014	★★★		★	★★	
Adriana	2018	★★★		★★	★★★	

Randomized trials were assessed by the method of Cochrane Handbook 5.1. Retrospective studies were assessed by NOS method. A study can be awarded a maximum of one star for each numbered item within the Selection and Outcome categories. A maximum of two stars can be given for Comparability, according to the instruction of NOS. *Abbreviation*: *Y* yes, *N* no, *NR* not reported

### Results of meta-analysis

#### Overall survival

**Fig. 2:**
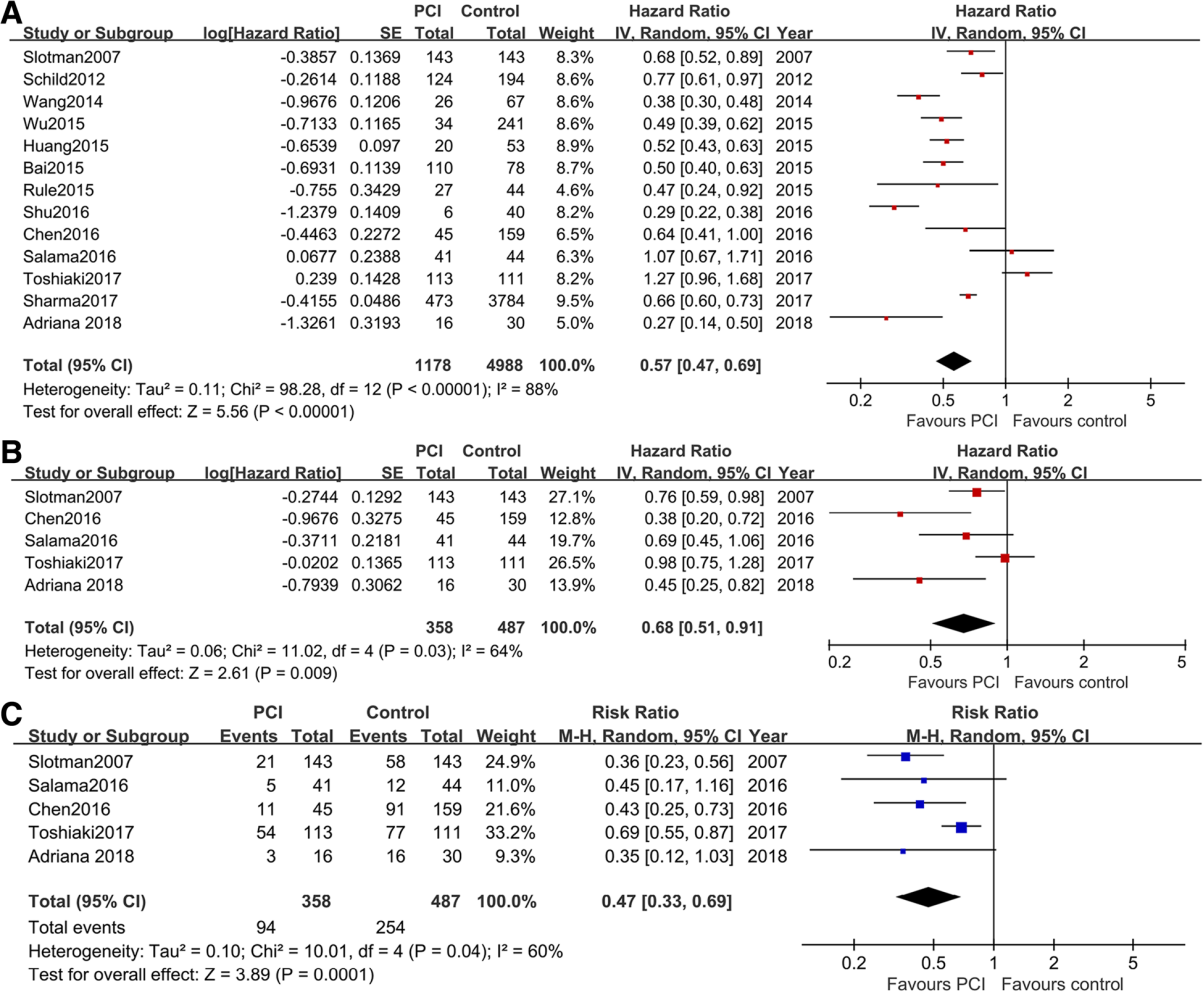
Combined effects of PCI versus control with regard to OS, PFS, and risk of brain metastasis. **a**, Forest plot showing the synthesized HR of PCI vs. control for OS in extensive-stage SCLC; **b**, Forest plot showing the synthesized HR of PCI vs. control for PFS in extensive-stage SCLC; **c**, PCI reduces the risk of brain metastasis in extensive-stage SCLC

HRs of PCI versus control for OS were extracted from 13 studies [7–10, 17, 18, 20–26]. As indicated by the I^2^ value of 88%, there was a significant heterogeneity between the included studies. So the random effect model was applied. As shown in Fig. 2a, the pooled analysis suggested that patients treated with PCI had a significantly better OS than that of the control group (HR = 0.57; 95% CI: 0.47, 0.69; *P* < 0.001). The reasons for heterogeneity may be related to different baseline characteristics, radiation dose, responses after chemotherapy, the number of included cases, and study quality.

#### Progression-free survival

Five studies [7, 10, 18, 20, 25] reported progression-free survival (PFS) in patients receiving PCI versus control. The random effect model was used as the I^2^ was 64%. As presented in Fig. 2b, application of PCI was associated with an improvement in PFS when compared to control (HR = 0.68; 95%CI: 0.51, 0.91; *p* = 0.009).

#### Incidence of brain metastases

Five studies [7, 10, 18, 20, 25] reported the incidence of brain metastases after PCI. The estimated RR of PCI versus control was 0.47 (95% CI: 0.33, 0.69; *P* = 0.0001), indicating that PCI was associated with less risk of BM (Fig. 2c).

#### Subgroup analysis

Next, we performed subgroup analysis to determine the impact of different factors on OS. These factors included study type, region, and PCI administration timing.

#### Study type

**Fig. 3:**
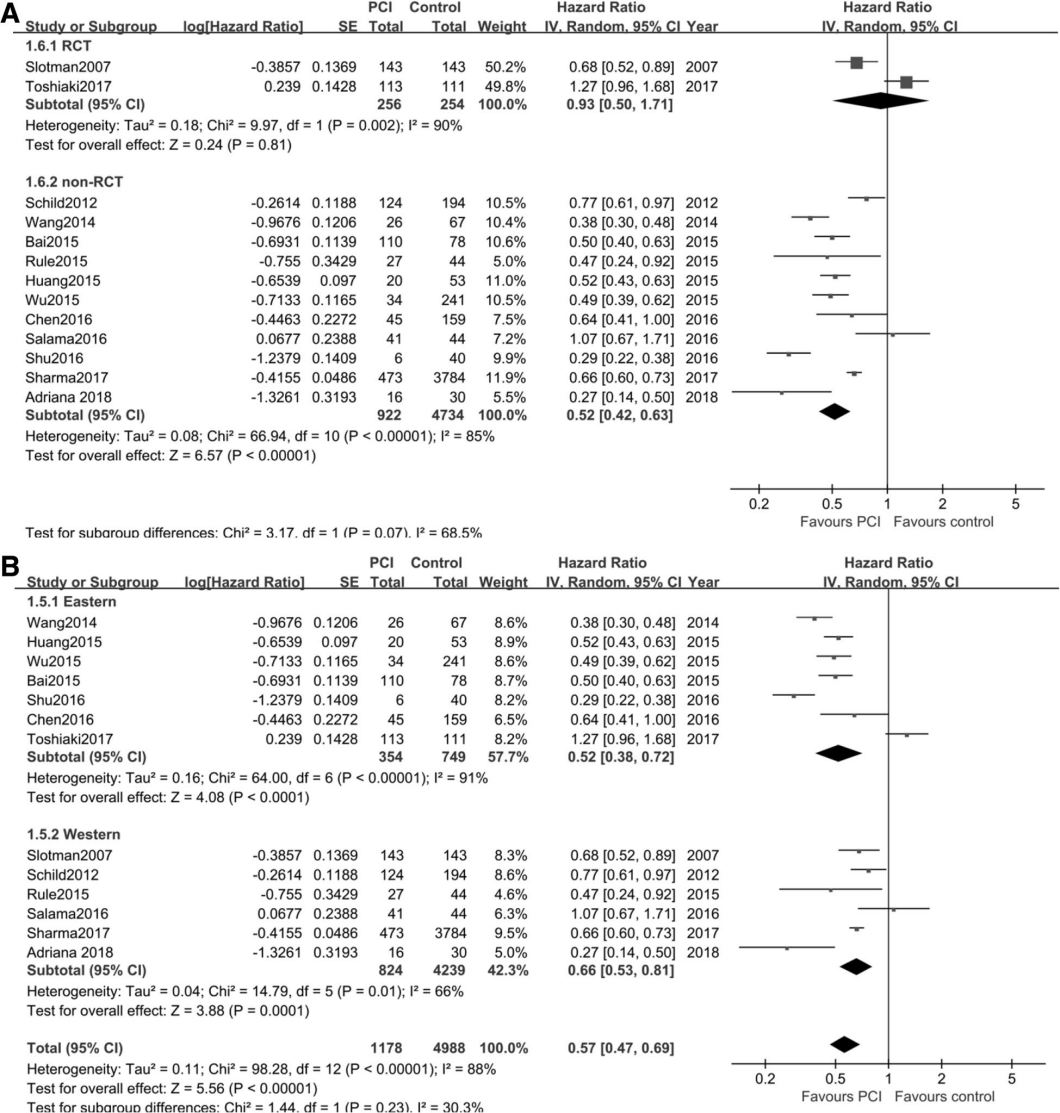
Forest plot of subgroup analysis and funnel plot on OS. **a**, subgroup analysis of study type; **b**, subgroup analysis of the region

There were two randomized controlled trials (RCT) [7, 10] and 11 retrospective studies [8, 9, 17–26]. We assessed the effect of PCI versus control on OS with regard to study type. Random effect model was applied to the overall analysis (I^2^ = 90% for RCT, and I^2^ = 85% for retrospective studies). The synthesized HR from two RCT was 0.93 (95% CI: 0.50, 1.71; *P* = 0.81), whereas it was 0.52 (95%CI: 0.42, 0.63; *P* < 0.01) from the retrospective studies (Fig. 3a).

#### Region

According to the sources of patients, the studies were classified as Eastern [10, 17, 20–23, 26] and Western [7–9, 18, 19, 24, 25]. As indicated by I^2^, a significant heterogeneity was detected and the random effect model was used. The combined HR of PCI versus control for OS in Eastern patients was 0.52 (95% CI: 0.38, 0.72; *P* < 0.01), and it was 0.66 (95% CI: 0.53, 0.81; *P* < 0.01) for Western patients (Fig. 3b).

#### PCI administration timing

**Fig. 4:**
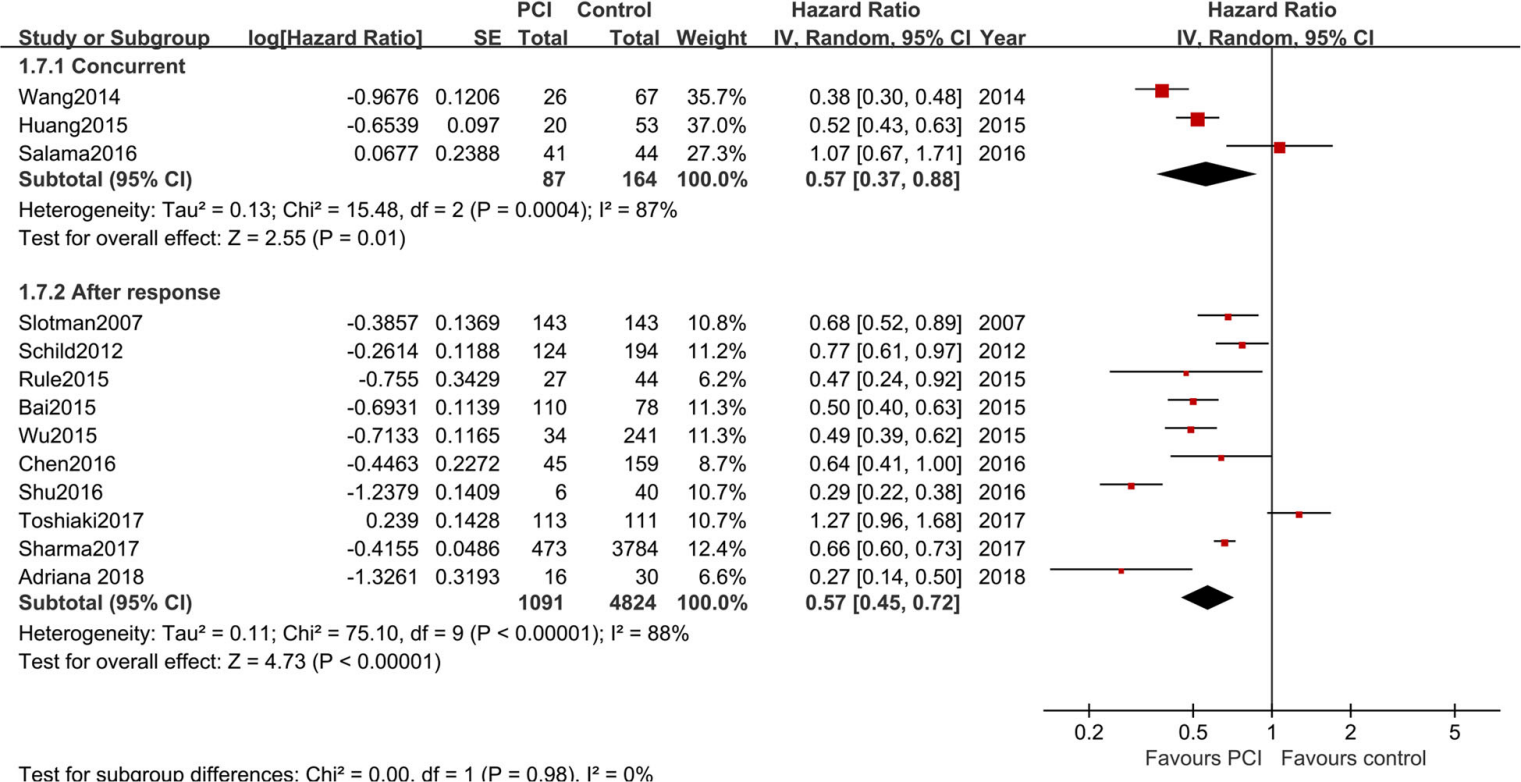
Subgroup analysis of PCI timing in extensive-stage SCLC

Ten studies used PCI to treat patients who responded to previous chemotherapy [7–10, 17, 20, 22–25], and four studies used concurrent chemotherapy and PCI [18, 19, 21, 26] to treat extensive-stage SCLC. As shown in Fig. 4, similar HRs were found for both subgroups (*P* < 0.05 for all).

#### Detection of publication bias and sensitivity analysis

We used the funnel plot, Begg’s and Egger’s method to test publication bias. As shown by the funnel plot, no obvious asymmetry was found in term of OS (Additional file 1: Figure S1A). A *P* of 0.76 obtained by Begg’s test and a *P* of 0.551 obtained by Egger’s test all suggested that there was no significant publication bias. The sensitivity analysis (Additional file 1: Figure S1B) was performed to test the stability of the findings. The results suggested that the effects of PCI on OS and BM incidence were reliable.

## Discussion

In this meta-analysis, 14 clinical trials were included. The combined results showed that PCI improved OS and reduced incidences of BM in extensive-stage SCLC. Subgroup analyses of OS suggested that the benefits of PCI in improving OS were independent of region, and PCI administration timing.

The combined results found that PCI had the ability to prolong OS, while the 1-year, 2-year and 3-year survival rates (data not shown) were similar between PCI versus control. These findings were thought to be controversial. One explanation was the limited number of included studies. Although most of the studies provided OS, three studies exhibited survival rate. Another reason may be the differences in study design. The objectives of the included randomized trials were different. The primary objective of Slotman et al. [7] was to determine whether PCI could reduce the incidence of symptomatic BM, whereas Toshiaki et al. [10] aimed to compare the efficacy of PCI on OS in BM free patients (previously checked by imaging). This indicated selection bias probably existed among studies. A third reason may be that the HR stands for the overall hazard ratio of getting improvement in survival but not for single time-point. One time-point is not capable of estimating the overall effect of PCI on survival.

Recently, there was a meta-analysis [27] evaluating the impact of PCI in extensive-stage SCLC. They included five studies comprising 984 cases. They found a similar role of PCI in prolonging PFS and reducing the risk of BM. This was in accordance with our findings. However, their results showed PCI did not statistically prolong OS when compared to control (HR = 0.82; 95% CI: 0.60–1.11; *p* = 0.19), and the PCI group had a better 1-year survival (37.1% versus 27.1%; RR = 0.87; *p* = 0.002) compared to the control group [27]. In contrast, our analysis shows a statistical advantage of PCI on OS (HR = 0.57; 95%CI: 0.47, 0.69; *p* < 0.00001). However, we also observe no significant HR after analyzing data from the included RCTs, and the previously described different aims of these two studies may be responsible for this result.

There were several limitations within our study. First, only two randomized controlled trials were included and the others were retrospective studies. Retrospective studies could introduce more selection bias and selective reporting. Secondly, the baseline characteristics along with the treatment history and PCI dose varied, which may be the origins of heterogeneity across included studies. The baseline characteristics of included studies indicated that the source of heterogeneity could be the region, chemotherapy response history, and timing of PCI. Other features like age, gender, performance status, PCI dose, and radiation technology may also contribute to the heterogeneity. These differences did not significantly affect the effect of PCI on OS as indicated by the sensitivity analysis. Thirdly, some of the HRs were indirectly collected which may underestimate or overestimate the actual role of PCI on survival. Forth, though there was no language limitation, the search language used in this study was only English and Chinese. Other language articles were not included in the study, which may cause unavoidable bias. Finally, although our study did not find obvious publication bias, it could not be completely avoided. This was because positive research results were more likely to be published, and negative research results were more likely to be rejected or not published.

## Conclusion

Our findings suggest that PCI improves survival and reduces brain metastasis in extensive-stage SCLC patients. However, more randomized controlled trials are needed to verify our findings.

## Additional file

Additional file 1: **Figure S1.** Funnel plot and sensitivity analysis on OS. A, funnel plot for OS; B, sensitivity analysis of PCI vs. control for OS in extensive-stage SCLC (TIF 1370 kb)

## Abbreviations

BM: brain metastasis
CBM: Chinese Biomedical Literature database
CI: confidence interval
DFS: disease free survival
EC: etoposide and carboplatin
EP: etoposide and cisplatin
F: female
HR: hazard ratio
IP: Irinotecan and cisplatin
M: male
N: no
N: number
NA: not available
NOS: Newcastle-Ottawa scale
NR: not reported
ORR: overall response rate
OS: overall survival
PCI: prophylactic cranial irradiation
PFS: progression-free survival
RCT: randomized controlled trials
RR: risk ratio
SCLC: small-cell lung cancer
SR: survival rate
Y: yes
