# Supplementary figures and images for: The effects of prophylactic cranial irradiation versus control on survival of patients with extensive-stage small-cell lung cancer: a meta-analysis of 14 trials
Source: Radiat Oncol. 2018 Aug 23;13:155. doi: 10.1186/s13014-018-1101-3 (PMC6107943; doi:10.1186/s13014-018-1101-3)

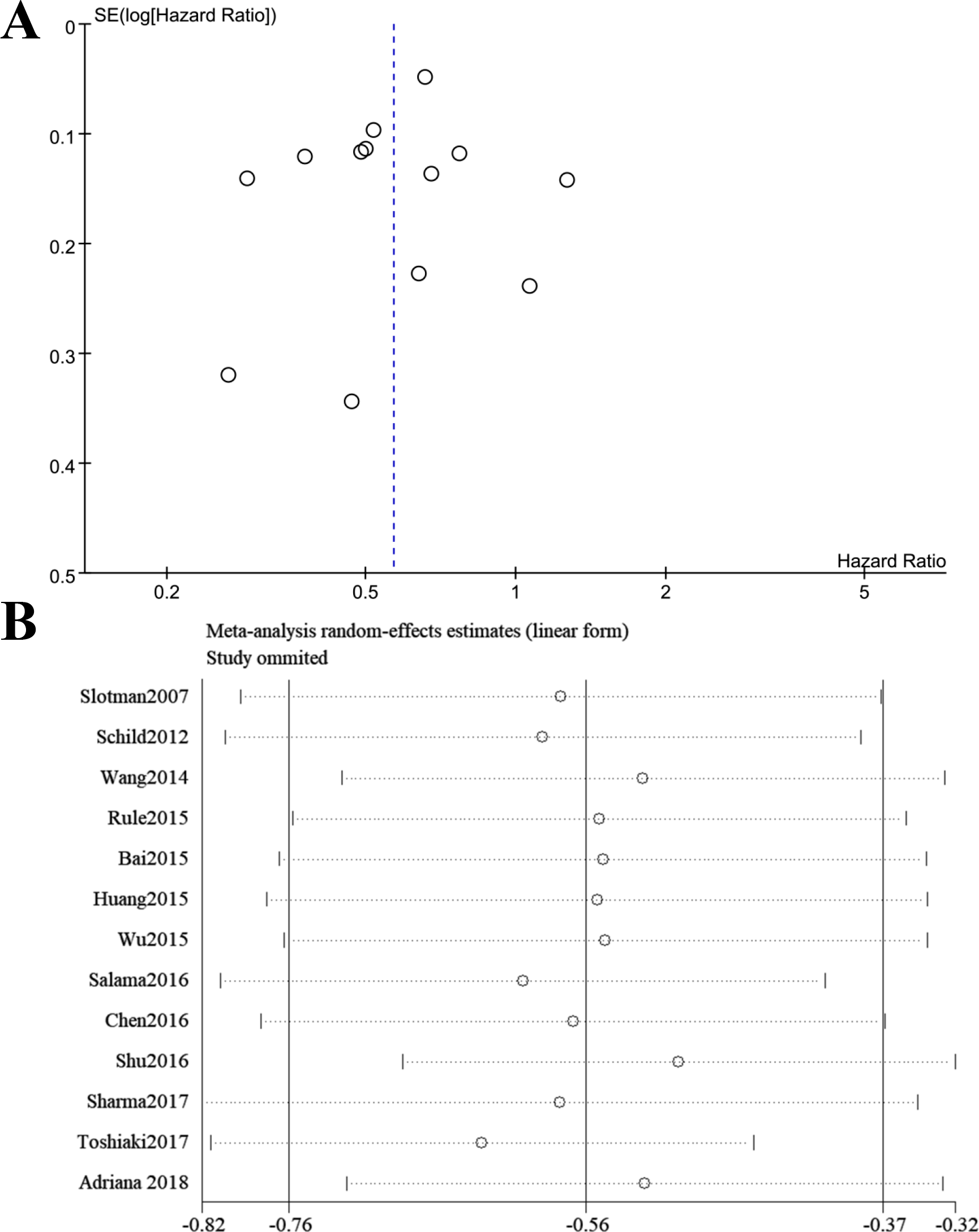

Supplement: Supplementary file 1 — Figure S1. Funnel plot and sensitivity analysis on OS. A, funnel plot for OS; B, sensitivity analysis of PCI vs. control for OS in extensive-stage SCLC (TIF 1370 kb) [file 13014_2018_1101_MOESM1_ESM.tif]
